# Supplementary material for: Dynamically adaptive soft metamaterial for wearable human–machine interfaces
Source: Nat Commun. 2025 Mar 19;16:2621. doi: 10.1038/s41467-025-57634-8 (PMC11923287; doi:10.1038/s41467-025-57634-8)
Supplement: Supplementary file 1 — Supplementary Information [file 41467_2025_57634_MOESM1_ESM.pdf]

Dynamically Adaptive Soft Metamaterial for Wearable Human-Machines Interfaces

Ugur Tanriverdi[1,2†], Guglielmo Senesi[1,2†], Tarek Asfour[1,2†], Hasan Kurt[1], Sabrina L. Smith[1,3], Diana Toderita[1], Joseph Shalhoub[4,5], Laura Burgess[6], Anthony Bull[1], Firat Güder[1*]

[1] Department of Bioengineering, Imperial College London, London, SW7 2AZ, UK.

[2] Unhindr Ltd, Burford Road, London, E15 2SP, UK.

[3] Barts and the London School of Medicine and Dentistry, Queen Mary University of London, London, E1 2AD, UK.

[4] Imperial Vascular Unit, Imperial College Healthcare NHS Trust, St Mary's Hospital, London W2 1NY, UK.

[5] Department of Surgery and Cancer, Imperial College London, London, SW7 2AZ, UK

[6] Charing Cross Hospital, Imperial College Healthcare NHS Trust, London, W6 8RF, UK.

[†] These authors contributed equally to this work: Ugur Tanriverdi, Guglielmo Senesi, Tarek Asfour.

* Corresponding author. Email: guder@ic.ac.uk

Experimental Details

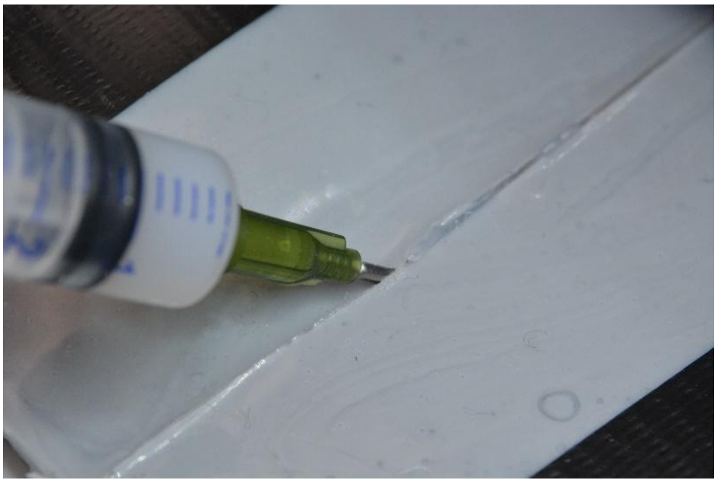

Fig. S1. The Roliner cylinder closure was realized using with Silpoxy glue. The application of Silpoxy glue between the unmet ends of the Roliner cylinder was performed using a blunt-end syringe underneath a sacrificial adhesive tape.

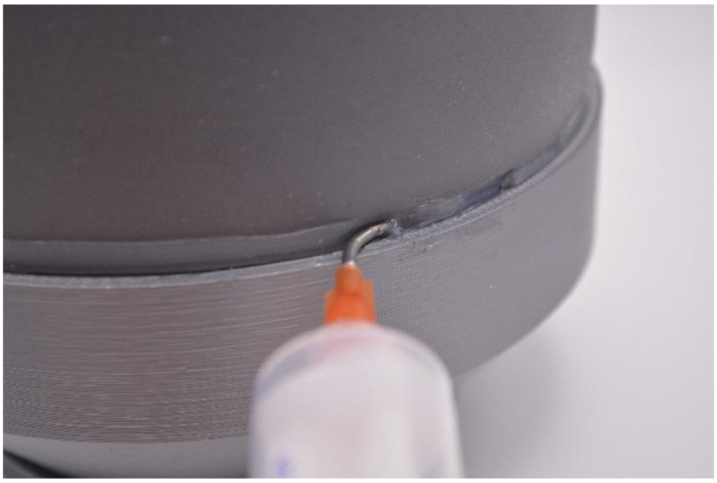

Fig. S2. The bottom dome of the Roliner is sealed using again Silpoxy glue. The glue was applied using a blunt-end bent syringe while the dome is pressure fitted into the reconfigurable composite.

Tensile Elasticity (TE)

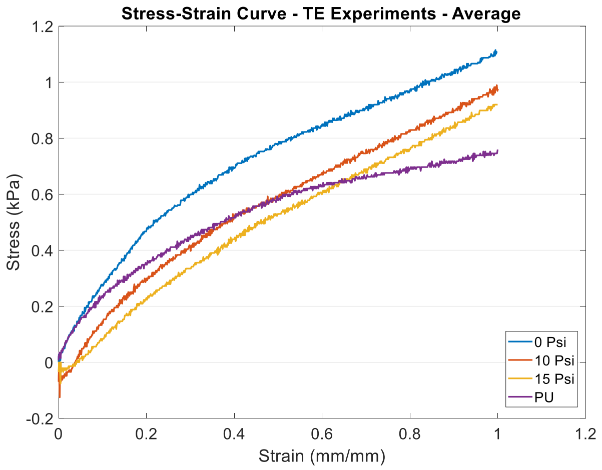

Fig. S3. The average stress vs strain graph of specimens used in tensile elasticity test. The tensile elasticity test is described in Fig. 3 in the main article. The reconfigurable composite samples which are actuated with actuation pressure of 0, 10, and 15 psi in comparison to bare polyurethane (PU) sample.

Compressive Elasticity (CE)

Table S1. The batch and specimen information of samples used in compressive elasticity tests in Fig. 3. Three different batches and six specimens in each batch are evaluated in terms of contact load (N) at thickness measurement and thickness (mm). The thickness of each specimen was measured and recorded individually. The average values are presented in the last column of the table.

**Batch #1**	**Specimen #1**	**Specimen #2**	**Specimen #3**	**Specimen #4**	**Specimen #5**	**Specimen #6**	**Average**
Contact Load at thickness measurement (N)	3.3	3.3	3.2	4.1	3.2	3.3	3.40
Thickness (mm)	6.68	6.52	6.7	6.58	6.56	6.71	6.63
**Batch #2**	**Specimen #1**	**Specimen #2**	**Specimen #3**	**Specimen #4**	**Specimen #5**	**Specimen #6**	-
Contact Load at thickness measurement (N)	3.2	3	2.7	3.8	3.8	2.4	3.15
Thickness (mm)	6.57	6.66	6.59	6.69	6.63	6.61	6.63
**Batch #3**	**Specimen #1**	**Specimen #2**	**Specimen #3**	**Specimen #4**	**Specimen #5**	**Specimen #6**	-
Contact Load at thickness measurement (N)	2.7	3.6	2.6	3.9	2.6	3.3	3.12
Thickness (mm)	6.74	6.41	6.65	6.69	6.69	6.64	6.64

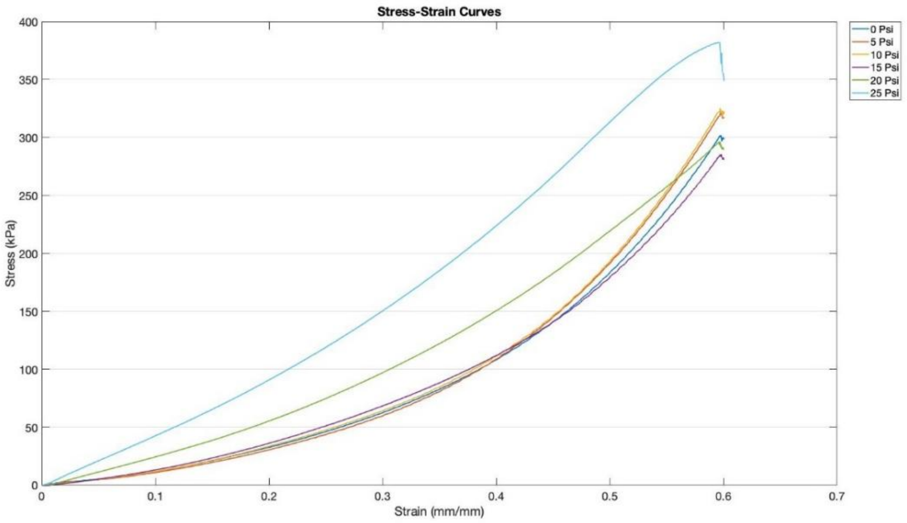

Fig. S4. Stress vs strain graph of all specimens in compressive elasticity test. The average stress vs strain graph of specimens used in compressive elasticity test. The compressive elasticity test is described in Fig. 3. in the main article. The reconfigurable composite samples which are actuated with actuation pressure of 0 – 25 psi.

Volumetric Elasticity (VE)

Table S2. The batch and specimen information of samples used in volumetric elasticity tests in Fig. 3. Three different batches and six specimens in each batch are evaluated in terms of contact load (N) at thickness measurement and thickness (mm). The thickness of each specimen was measured and recorded individually. The average values are presented in the last column of the table.

**Batch #1**	**Specimen #1**	**Specimen #2**	**Specimen #3**	**Specimen #4**	**Specimen #5**	**Specimen #6**	**Average**
Contact Load at thickness measurement (N)	3.5	2.4	3.5	3.6	3	3.3	3.22
Thickness (mm)	6.49	6.43	6.72	6.47	6.71	6.53	6.56
**Batch #2**	**Specimen #1**	**Specimen #2**	**Specimen #3**	**Specimen #4**	**Specimen #5**	**Specimen #6**	**-**
Contact Load at thickness measurement (N)	3.9	3.8	2.9	3	3.9	3.3	3.47
Thickness (mm)	6.5	6.56	6.64	6.68	6.73	6.78	6.65
**Batch #3**	**Specimen #1**	**Specimen #2**	**Specimen #3**	**Specimen #4**	**Specimen #5**	**Specimen #6**	**-**
Contact Load at thickness measurement (N)	3.3	3.9	3.2	3.2	3	3.5	3.35
Thickness (mm)	6.63	6.72	6.63	6.76	6.5	6.5	6.62

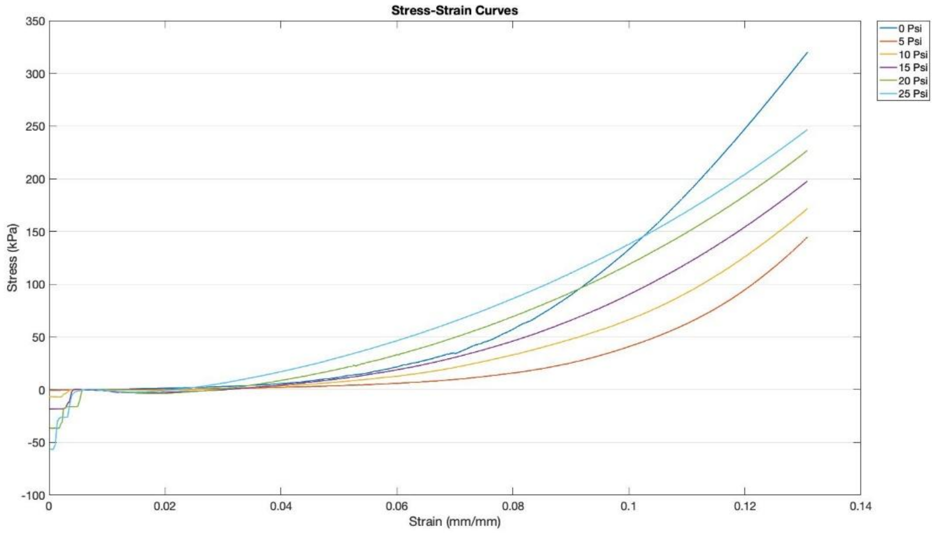

Fig. S5. Stress vs strain graph of all specimens in volumetric elasticity test. The average stress vs strain graph of specimens used in volumetric elasticity test. The volumetric elasticity test is described in Fig. 3. in the main article. The reconfigurable composite samples which are actuated with actuation pressure of 0 – 25 psi.

Liner Characterization

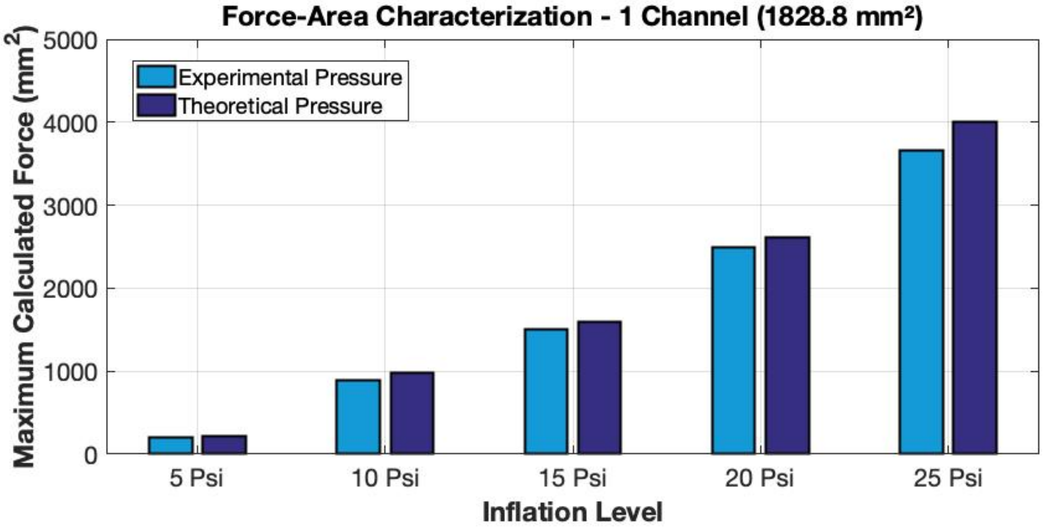

Fig. S6. Force-Area characterization of Roliner with a single channel actuated configuration at different actuation (inflation) pressure levels between 5 – 25 psi (with 5 psi intervals). The single channel constitutes an area of 1828.8 mm^2 actuated area in the Roliner. The experimental and theoretical pressure values were represented with blue- and purple-colored bars, respectively.

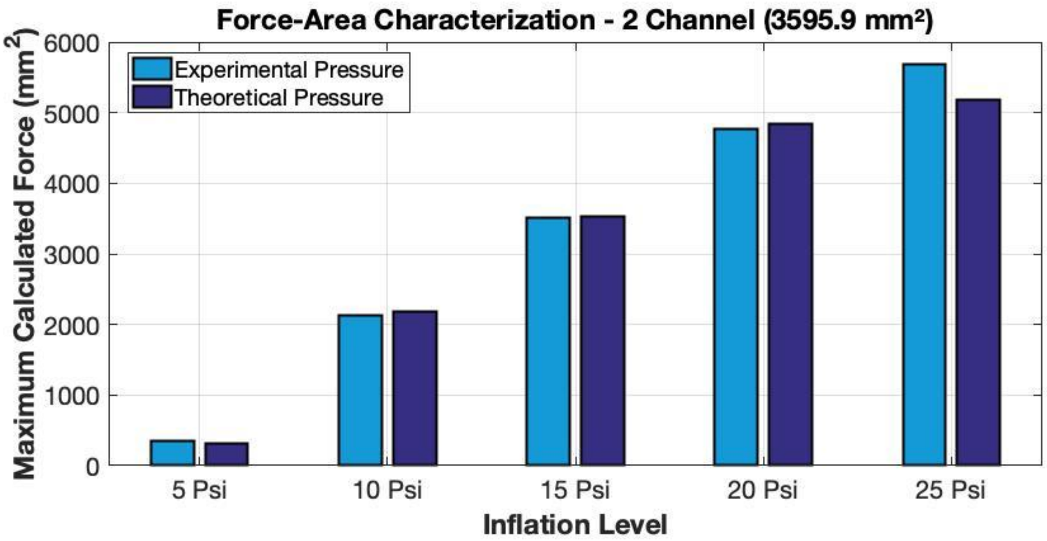

Fig. S7. Force-Area characterization of Roliner with two-channel actuated configuration at different actuation (inflation) pressure levels between 5 – 25 psi (with 5 psi intervals). The single channel constitutes an area of 3595.9 mm^2 actuated area in the Roliner. The experimental and theoretical pressure values were represented with blue- and purple-colored bars, respectively.

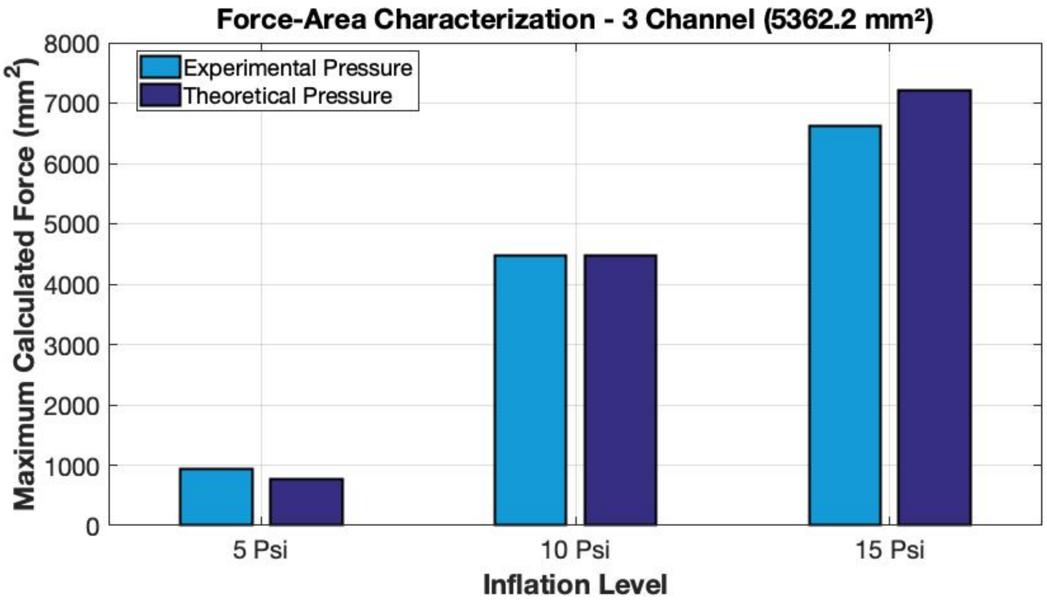

Fig. S8. Force-Area characterization of Roliner with three-channel actuated configuration at different actuation (inflation) pressure levels between 5 – 15 psi (with 5 psi intervals). The single channel constitutes an area of 5362.2 mm^2 actuated area in the Roliner. The experimental and theoretical pressure values were represented with blue and purple colored bars, respectively.

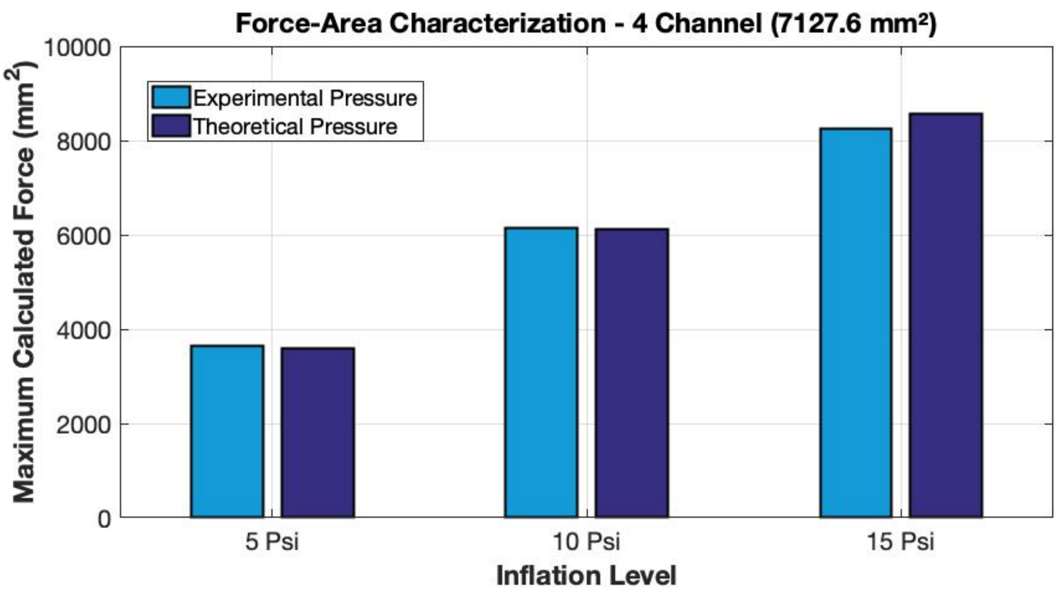

Fig. S9. Force-Area characterization of Roliner with four-channel (all) actuated configuration at different actuation (inflation) pressure levels between 5 – 15 psi (with 5 psi intervals). The single channel constitutes an area of 7127.6 mm^2 actuated area in the Roliner. The experimental and theoretical pressure values were represented with blue and purple colored bars, respectively.

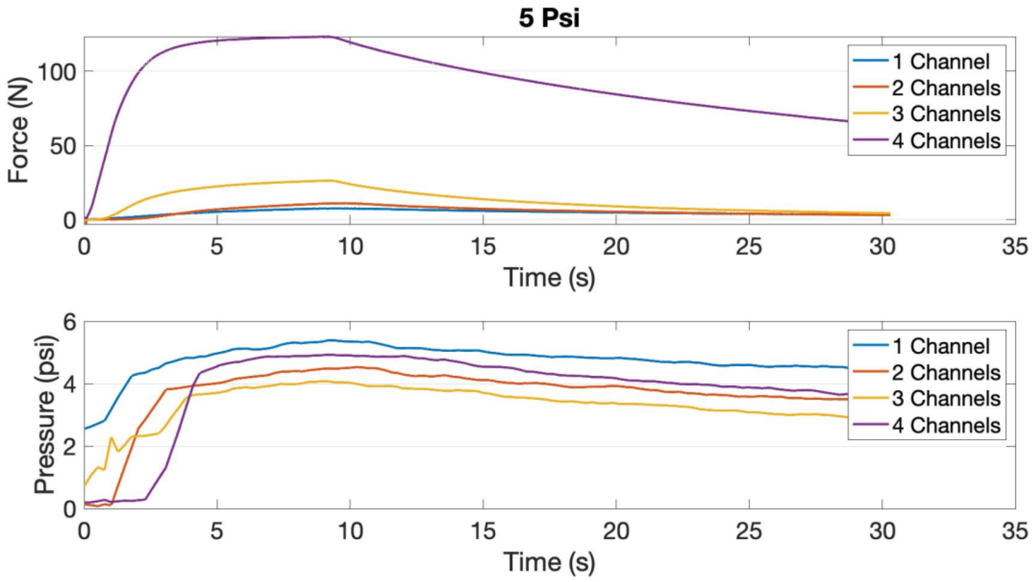

Fig. S10. The force exerted by Roliner when actuated with a pressure of 5 psi in 1-4 channel configurations is presented in a period of 30 seconds. (top) The measured fluidics pressure exerted by Roliner when actuated with a pressure of 5 psi in 1-4 channel configurations is presented in a period of 30 seconds. (bottom). The maximum points were used in Fig. 4. of the main article.

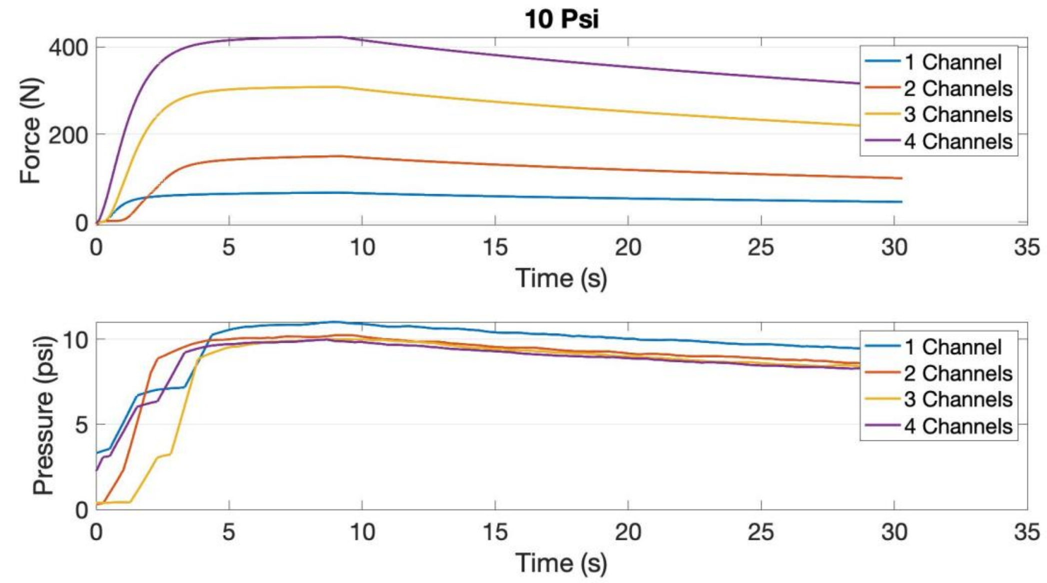

Fig. S11. The force exerted by Roliner when actuated with a pressure of 10 psi in 1-4 channel configurations is presented in a period of 30 seconds. (top) The measured fluidics pressure exerted by Roliner when actuated with a pressure of 10 psi in 1-4 channel configurations is presented in a period of 30 seconds. (bottom). The maximum points were used in Fig. 4. of the main article.

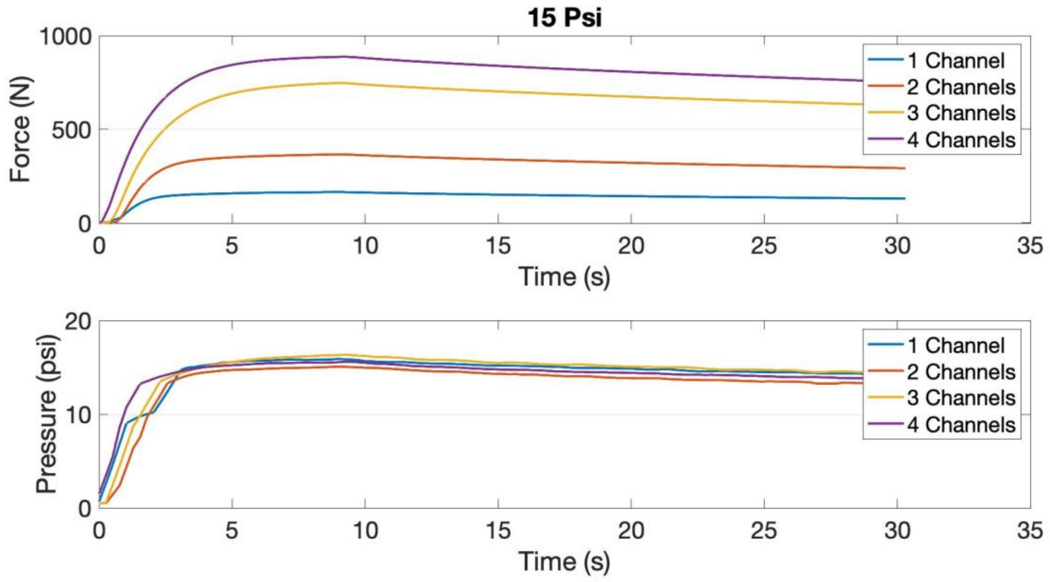

Fig. S12. The force exerted by Roliner when actuated with a pressure of 15 psi in 1-4 channel configurations is presented in a period of 30 seconds. (top) The measured fluidics pressure exerted by Roliner when actuated with a pressure of 15 psi in 1-4 channel configurations is presented in a period of 30 seconds. (bottom). The maximum points were used in Fig. 4. of the main article.

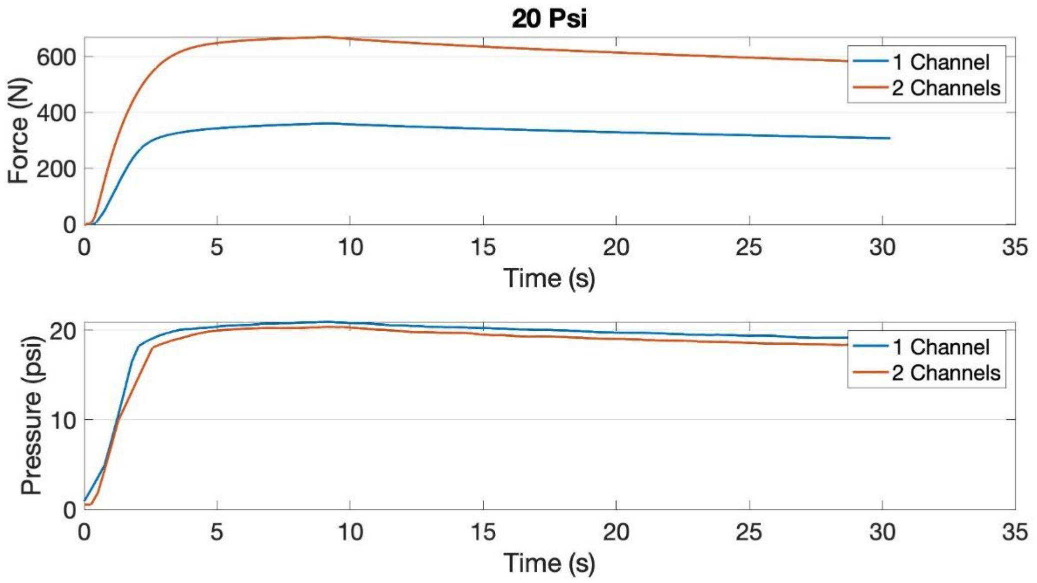

Fig. S13. The force exerted by Roliner when actuated with a pressure of 20 psi in 1-2 channel configurations is presented in a period of 30 seconds. (top) The measured fluidics pressure exerted by Roliner when actuated with a pressure of 20 psi in 1-2 channel configurations is presented in a period of 30 seconds. (bottom). The maximum points were used in Fig. 4. of the main article.

Gait Cycle Analysis

The definition of the tracked parameters in gait cycle analysis are listed below:

Stance/Swing Time: The duration a foot is in contact with the ground during a gait cycle, while swing time denotes the period the foot is off the ground. Increased stance time might indicate impaired balance and muscle or joint weakness, leading to a more cautious gait.

Vertical Ground Reaction Force (GRF): Vertical GRF measures the force exerted by the ground on the body in the vertical direction during walking or running. Lower GRF might be seen in walking or in individuals with softer gait patterns to minimize joint loading or in amputees who don't trust their prosthetic leg.

Cadence: The number of steps taken per minute, is a fundamental parameter of gait speed and rhythmicity. Higher cadence is typically associated with a quicker, more efficient walking or running pace, often seen in athletes. Lower cadence may indicate fatigue, reduced mobility, or balance issues. Lower cadence, however, can also be observed in experimental setups or devices where the user is not accustomed to the environment.

Stride Length/Width: Stride length is the distance between successive placements of the same foot, while stride width is the lateral distance between the feet during walking. Correlated with cadence, increased stride length is often associated with faster walking speeds and better overall gait efficiency, typical in younger, healthy adults. Increased stride width, on the other hand, may suggest a compensatory mechanism for balance, which is common in individuals with cerebellar ataxia.

Range of Motion (ROM): The range of motion for the ankle, hip, and knee joints during gait is critical for evaluating joint flexibility and functionality. Greater ROM in these joints generally reflects better joint health and flexibility, which is essential for efficient gait. Reduced ROM, such as limited ankle

dorsiflexion, can impair gait patterns and is often observed in conditions like stroke or joint contractures, leading to a shuffling gait.

Self-Selected Speed: Self-selected speed is the walking speed that an individual chooses naturally. Higher self-selected speed indicates better functional mobility and cardiovascular fitness, commonly seen in healthy adults. Lower self-selected speed is often a sign of decreased functional capacity and can be predictive of frailty and increased fall risk, frequently observed in older adults or those with chronic conditions. Similar to cadence, self-selected speed can decrease when walking in an unfamiliar environment with unfamiliar wearable gear.

Table S3. The gait cycle analysis of prosthesis user SJ03. The user SJ03 had left leg transfemoral amputation. The gait cycle was tracked while the user was equipped with user's own passive liner (as control), and Roliner in both passive and active (actuated) forms. The description of the parameters was represented in above section. The column named "sound" designates the sound leg. The column named "prosthetic" designates the amputated leg.

	**Control**				**Roliner - Passive**				**Roliner - Active**			
	Sound		Prosthetic		Sound		Prosthetic		Sound		Prosthetic	
**Parameters**	Mean	SD	Mean	SD	Mean	SD	Mean	SD	Mean	SD	Mean	SD
Cadence (step/min)	**49.83**	2.01	**54.22**	6.95	**52.2**	3.43	**55.69**	7.52	**54.06**	6.62	**51.64**	1.39
Vertical GRF - 1st Peak (N/kg)	**N/A**		**N/A**		**N/A**		**N/A**		**N/A**		**N/A**	
Vertical GRF - 2nd Peak (N/kg)	**N/A**		**N/A**		**N/A**		**N/A**		**N/A**		**N/A**	
Hip ROM (deg)	**25.27**	27.72	**36.64**	0.66	**25.7**	28.17	**35.7**	1.55	**39.34**	22.04	**35.05**	1.37
Knee ROM (deg)	**32.4**	35.49	**63.05**	0.68	**33.87**	37.13	**63.9**	1.25	**49.38**	27.88	**63.49**	0.72
Ankle ROM (deg)	**16.56**	18.16	**11.07**	0.16	**16.15**	17.77	**11.27**	0.56	**25.19**	14.13	**10.91**	0.09
Stance time (% of gait cycle)	**68.99**	1.49	**57.63**	5.74	**65.06**	2.8	**55.00**	6.51	**66.24**	4.15	**59.74**	1.2
Swing time (% of gait cycle)	**31.01**	1.49	**42.37**	5.74	**34.93**	2.8	**45.00**	6.51	**33.76**	4.15	**40.25**	1.2
Stride Length (m)	**1.28**	0.32	**1.58**	0.06	**1.16**	0.51	**1.58**	0.06	**1.47**	0.2	**1.57**	0.03
Stride Width (mm)	**44.27**	40.2	**45.55**	15.27	**31.82**	12.99	**66.45**	21.72	**58.29**	24.2	**56.48**	12.83
Pistoning (mm)	**N/A**		**38.22**	1.8	**N/A**		**41.33**	2.48	**N/A**		**39.2**	2.77
Self Selected Speed (km/h)	**4.314**				**4.7049**				**3.3932**			

Table S4. The gait cycle analysis of prosthesis user SJ04. The user SJ04 had left leg transfemoral amputation. The gait cycle was tracked while the user was equipped with user's own passive liner (as control), and Roliner in both passive and active (actuated) forms. The description of the parameters was represented in above section. The column named "sound" designates the sound leg. The column named "prosthetic" designates the amputated leg.

	**Control**				**Roliner - Passive**				**Roliner - Active**			
	Sound		Prosthetic		Sound		Prosthetic		Sound		Prosthetic	
**Parameters**	Mean	SD	Mean	SD	Mean	SD	Mean	SD	Mean	SD	Mean	SD
Cadence (step/min)	**52.43**	1.79	**53.85**	1.54	**N/A**		**N/A**		**54.93**	5.10	**53.72**	2.20
Vertical GRF - 1st Peak (N/kg)	**12.04**	0.32	**10.44**	2.41	**N/A**		**N/A**		**12.04**	0.19	**12.76**	1.32
Vertical GRF - 2nd Peak (N/kg)	**10.75**	0.26	**9.99**	0.55	**N/A**		**N/A**		**10.26**	1.17	**10.61**	0.42
Hip ROM (deg)	**50.98**	0.81	**39.93**	1957	**N/A**		**N/A**		**44.95**	22.14	**45.13**	1.45
Knee ROM (deg)	**59.75**	1.46	**55.73**	27.31	**N/A**		**N/A**		**47.86**	27.07	**67.18**	1.23
Ankle ROM (deg)	**43.07**	1.42	**17.15**	8.43	**N/A**		**N/A**		**35.79**	18.04	**22.66**	1.62
Stance time (% of gait cycle)	**64.27**	1.76	**62.33**	2.44	**N/A**		**N/A**		**65.47**	4.97	**59.54**	2.23
Swing time (% of gait cycle)	**35.72**	1.76	**37.66**	2.44	**N/A**		**N/A**		**34.52**	4.97	**40.45**	2.23
Stride Length (m)	**1.51**	0.34	**1.50**	0.03	**N/A**		**N/A**		**1.22**	0.46	**1.53**	0.04
Stride Width (mm)	**55.57**	25.77	**77.70**	25.32	**N/A**		**N/A**		**61.94**	25.99	**74.84**	18.74
Pistoning (mm)	**N/A**		**113.41**	4.72	**N/A**		**N/A**		**N/A**		**116.12**	4.93
Self Selected Speed (km/h)	**4.4969**				**N/A**				**3.696**			

Table S5. The gait cycle analysis of prosthesis user SJ05. The user SJ05 had left leg transfemoral amputation. The gait cycle was tracked while the user was equipped with user's own passive liner (as control), and Roliner in both passive and active (actuated) forms. The description of the parameters was represented in above section. The column named "sound" designates the sound leg. The column named "prosthetic" designates the amputated leg.

	**Control**				**Roliner - Passive**				**Roliner - Active**			
	Sound		Prosthetic		Sound		Prosthetic		Sound		Prosthetic	
**Parameters**	Mean	SD	Mean	SD	Mean	SD	Mean	SD	Mean	SD	Mean	SD
Cadence (step/min)	**53.17**	1.48	**51.89**	0	**53.33**	2.50	**53.58**	0.28	**53.76**	1.19	**53.05**	0.62
Vertical GRF - 1st Peak (N/kg)	**10.71**	0.46	**11.23**	0.33	**9.91**	1.44	**9.03**	4.16	**11.06**	0.47	**11.33**	0.50
Vertical GRF - 2nd Peak (N/kg)	**10.50**	1.33	**10.45**	0.16	**8.81**	1.75	**8.97**	4.12	**10.32**	0.50	**10.52**	0.36
Hip ROM (deg)	**38.19**	21.39	**42.49**	1.09	**46.97**	2.27	**30.47**	20.32	**33.26**	28.81	**41.40**	0.81
Knee ROM (deg)	**50.17**	28.14	**67.19**	2.20	**61.26**	1.76	**50.05**	33.45	**20.97**	36.33	**67.08**	1.93
Ankle ROM (deg)	**33.00**	11.08	**6.72**	6.14	**36.57**	1.70	**6.93**	8.27	**12.21**	21.16	**7.46**	6.46
Stance time (% of gait cycle)	**63.48**	1.7818	**63.06**	0	**63.90**	0.78	**61.90**	0.26	**65.49**	1.23	**60.65**	2.72
Swing time (% of gait cycle)	**36.51**	1.7818	**36.93**	0	**36.09**	0.78	**38.09**	0.26	**34.50**	1.23	**39.34**	2.72
Stride Length (m)	**0.97**	0.51	**1.21**	0.01	**1.20**	0.03	**1.10**	0.22	**0.85**	0.39	**1.22**	0.02
Stride Width (mm)	**45.47**	21.1593	**66.27**	35.25	**56.22**	23.23	**70.66**	29.63	**44.07**	18.37	**62.45**	17.27
Pistoning (mm)	**N/A**		**48.01**	2.64	**N/A**		**44.86**	1.11	**N/A**		**41.32**	3.75
Self Selected Speed (km/h)	**3.944**				**3.8572**				**3.6359**			

Table S6. The gait cycle analysis of prosthesis user SJ06. The user SJ06 had right leg transfemoral amputation. The gait cycle was tracked while the user was equipped with user's own passive liner (as control), and Roliner in both passive and active (actuated) forms. The description of the parameters was represented in the above section. The column named "sound" designates the sound leg. The column named "prosthetic" designates the amputated leg.

	**Control**				**Roliner - Passive**				**Roliner - Active**			
	Sound		Prosthetic		Sound		Prosthetic		Sound		Prosthetic	
**Parameters**	Mean	SD	Mean	SD	Mean	SD	Mean	SD	Mean	SD	Mean	SD
Cadence (step/min)	**51.42**	1.93	**51.13**	1.97	**51.02**	0.94	**50.40**	1.12	**49.48**	2.14	**51.29**	1.65
Vertical GRF - 1st Peak (N/kg)	**12.10**	1.11	**10.26**	0.34	**11.44**	0.48	**10.49**	0.22	**11.62**	0.39	**10.72**	0.32
Vertical GRF - 2nd Peak (N/kg)	**10.42**	0.43	**10.16**	0.36	**10.62**	0.31	**9.740**	0.24	**11.02**	0.36	**9.77**	0.14
Hip ROM (deg)	**39.80**	19.51	**40.32**	0.93	**46.92**	1.24	**37.89**	1.77	**32.85**	25.51	**7.14**	17.50
Knee ROM (deg)	**54.95**	26.4	**67.12**	1.48	**64.63**	1.20	**67.38**	1.68	**45.52**	36.53	**10.19**	24.96
Ankle ROM (deg)	**19.90**	9.77	**13.34**	2.39	**29.58**	2.96	**13.997**	1.95	**35.97**	33.94	**3.48**	8.52
Stance time (% of gait cycle)	**67.54**	1.4	**57.64**	0.82	**67.42**	1.02	**58.95**	1.26	**65.75**	3.57	**57.37**	1.29
Swing time (% of gait cycle)	**32.45**	1.4	**42.44**	0.82	**32.58**	1.02	**41.05**	1.26	**34.25**	3.57	**42.63**	1.29
Stride Length (m)	**1.25**	0.027	**1.27**	0.034	**1.25**	0.01	**1.28**	0.02	**1.29**	0.12	**1.25**	0.02
Stride Width (mm)	**66.36**	13.16	**45.36**	13.3	**59.01**	16.06	**48.36**	15.4	**95.3**	35.42	**58.21**	19.41
Pistoning (mm)	**N/A**		**N/A**		**N/A**		**N/A**		**N/A**		**N/A**	
Self Selected Speed (km/h)	**3.530**				**3.580**				**3.220**			

Pre-clinical Inclusion/Exclusion Criteria

This pre-clinical trial was approved by Research Governance and Integrity Team (RGIT) under Imperial College Research Ethics Committee process (ICREC Ref No: 21IC7315) on December 2nd, 2021.

Inclusion Criteria

The amputees are selected from diverse ethnic and social backgrounds. The amputee must:

- be a lower-limb amputee between 18 and 60 years.
- be a healthy adult with no other medical conditions,
- have been an amputee for more than 18 months, traumatic or surgically induced.

Exclusion Criteria

Members of vulnerable groups are excluded (e.g., those under 18, prisoners, those in a dependent relationship, the mentally ill). Additionally, the amputee must not:

- have had diabetic or other vascular-related amputations,
- be taking any medication or receiving any therapy for another existing condition.
- have any allergies to adhesive materials.
- have undergone surgery in the lower limbs 12 months prior to participation.
- be unable to speak English at a sufficient level to give consent to participate.
- be pregnant.
- be younger than 18 or older than 60 years of age.
- weigh above 120 kg in mass.

Table S7. The physical details of the participants in the study.

Subject Number	Sex	Age	Height (cm)	Weight (kg)	Amputation Side	Amputation Level	Prosthetic Use Experience (years)
SJ01	M	28	175	78	Bi-lateral	Through-knee	11
SJ02	M	29	192	77.8	Right Leg	Transfemoral	10
SJ03	M	43	197	115	Left Leg	Transfemoral	9
SJ04	M	34	183	107.9	Left	Transfemoral	15
SJ05	F	28	155	48	Left	Transfemoral	12
SJ06	M	58	172	76	Right	Transfemoral	11

Table S8. The list of prosthetic limbs used by the participants in this study.

Subject Number	Prosthetic Limb Weight (kg)	Knee Model	Liner Brand	Liner Type	Liner Size	Removable Seal	Seal Size	Liner Weight (kg)	Liner Profile
SJ01	N/A	Stubbies	Ossur 4Seal	Seal-in	N/A	No	N/A	N/A	Conical
SJ02	5.0	Ossur Rheo Knee XC	Ossur Iceross	Seal-in	34	Yes	34	0.70	Cylindrical
SJ03	5.8	Ottobock Genium	Ossur Iceross	Seal-in	36	Yes	47	0.80	Conical
SJ04	5.4	C-leg 4 Otto Bock	Ossur Iceross	Seal-in	34	No	N/A	0.85	Conical
SJ05	4.2	C-leg 4 Otto Bock	Ossur Iceross	Seal-in	32	Yes	35	0.70	Conical
SJ06	4.8	Ottobock Genium	Ossur Iceross	Seal-in	38	No	N/A	0.80	Conical

PEQ Questionnaire

1. **Over the past four weeks, rate the fit of your prosthesis.**

TERRIBLE EXCELLENT

2. **Over the past four weeks, rate the weight of your prosthesis.**

TERRIBLE EXCELLENT

3. **Over the past four weeks, rate your comfort while standing *when using your prosthesis*.**

TERRIBLE EXCELLENT

4. **Over the past four weeks, rate your comfort while sitting *when using your prosthesis*.**

TERRIBLE EXCELLENT

5. **Over the past four weeks, rate how often you felt off balance *while using your prosthesis*.**

ALL THE TIME NOT AT ALL

6. **Over the past four weeks, rate how much energy it took to use your prosthesis for as long as you needed it.**

COMPLETELY EXHAUSTING NONE AT ALL

7. **Over the past four weeks, rate the feel (such as the temperature and texture) of the prosthesis (sock, liner, socket) on your residual limb (stump).**

WORST POSSIBLE BEST POSSIBLE

8. **Over the past four weeks, rate the ease of putting on (donning) your prosthesis.**

TERRIBLE EXCELLENT

9. **Over the past four weeks, rate how much you sweat inside your prosthesis (in the sock, liner, socket).**

EXTREME AMOUNT NOT AT ALL

10. **Over the past four weeks, rate how smelly your prosthesis was at its worst.**

EXTREMELY SMELLY NOT AT ALL

11. **Over the past four weeks, rate how much of the time your residual limb was swollen to the point of changing the fit of your prosthesis.**

ALL THE TIME NEVER

12. **Over the past four weeks, rate any rash(es) that you got on your residual limb.**

EXTREMELY BOTHERSOME NOT AT ALL

OR check __ I had no rashes on my residual limb in the last month.

13. Over the past four weeks, rate any ingrown hairs (pimples) that were on your residual limb.

EXTREMELY BOTHERSOME NOT AT ALL

OR check __ I had no ingrown hairs on my residual limb in the last months.

14. Over the past four weeks, rate any blisters or sores that you got on your residual limb.

EXTREMELY BOTHERSOME NOT AT ALL

OR check __ I had no blisters or sores on my residual limb in the last months.

15. Over the past four weeks, rate how frequently you were frustrated with your prosthesis.

ALL THE TIME NEVER

16. If you were frustrated with your prosthesis at any time over the past month, think of the most frustrating event and rate how you felt at that time.

EXTREMELY FRUSTRATED NOT AT ALL

OR check __ I have not been frustrated with my prosthesis.

17. Over the past four weeks, rate your ability to walk *when using your prosthesis*.

CANNOT NO PROBLEM

18. Over the past four weeks, rate your ability to walk in close spaces *when using your prosthesis*.

CANNOT NO PROBLEM

19. Over the past four weeks, rate your ability to walk up stairs *when using your prosthesis*.

CANNOT NO PROBLEM

20. Over the past four weeks, rate how you have felt about being able to walk down stairs *when using your prosthesis*.

CANNOT NO PROBLEM

21. Over the past four weeks, rate your ability to walk up a steep hill *when using your prosthesis*.

CANNOT NO PROBLEM

22. Over the past four weeks, rate your ability to walk down a steep hill *when using your prosthesis*.

CANNOT NO PROBLEM

23. Over the past four weeks, rate your ability to walk on sidewalks and streets *when using your prosthesis.*

CANNOT NO PROBLEM

24. Over the past four weeks, rate your ability to walk on slippery surfaces (e.g. wet tile, snow, a rainy street, or a boat deck) *when using your prosthesis*.

CANNOT NO PROBLEM

Table S9. The prosthesis evaluation questionnaire (PEQ) scores of passive liners for questions related to prosthesis utility (questions 1-8) are tabulated below. The average score and standard deviation (StD) of each question are shown at the end of each question columns. The average score of each participant is shown at the end of each user column.

Utility (Standard)

Question #	1			2			3			4			5			6			7			8			Score
Parameter	N	D	S	N	D	S	N	D	S	N	D	S	N	D	S	N	D	S	N	D	S	N	D	S	
SJ01	75.5	97	78%	36	97	37%	92.5	97	95%	72.5	97	75%	91.5	97	94%	55	97	57%	62.5	97	64%	92.5	97	95%	74.5%
SJ02	44	97	45%	50	97	52%	49	97	51%	58	97	60%	45	97	46%	18.5	97	19%	41.5	97	43%	61.5	97	63%	47.4%
SJ03	87.5	96	91%	87.5	96	91%	87	96	91%	87	96	91%	87.5	96	91%	88.5	96	92%	83	96	86%	83	96	86%	90.0%
SJ03(2nd)	65.5	96.5	68%	84	96.5	87%	83	96.5	86%	54	96.5	56%	63.5	96.5	66%	53.5	96.5	55%	66.5	96.5	69%	86	96.5	89%	72.0%
SJ04	15	96	16%	12	96	13%	18.5	96	19%	3	96	3%	12.5	96	13%	17.5	96	18%	7	96	7%	15	96	16%	13.1%
SJ04(2nd)	9	93	10%	6.5	93	7%	23	93	25%	2	93	2%	31	93	33%	22	93	24%	11	93	12%	12	93	13%	15.7%
SJ05	39	96	41%	67	96	70%	60	96	63%	48	96	50%	58	96	60%	65	96	68%	32.5	96	34%	36	96	38%	52.8%
SJ06	29	96	30%	69	96	72%	74	96	77%	93	96	97%	69	96	72%	37	96	39%	66	96	69%	75	96	78%	66.7%
SJ07	88	93	95%	92	93	99%	84	93	90%	92	93	99%	93	93	100%	85	93	91%	74	93	80%	91	93	98%	94.0%
SJ08	44.5	91	49%	64	91	70%	46	91	51%	61	91	67%	84	91	92%	71	91	78%	43	91	47%	65	91	71%	65.7%
Average			52%			60%			65%			60%			67%			54%			51%			65%	59.2%
StD			29.9%			32.0%			27.8%			34.6%			29.1%			28.5%			27.3%			31.9%	27.6%

Table S10. The prosthesis evaluation questionnaire (PEQ) scores of Roliner for questions related to prosthesis utility (questions 1-8) are tabulated below. The average score and standard deviation (StD) of each question are shown at the end of each question columns. The average score of each participant is shown at the end of each user column.

Utility (Roliner)

Question #	1			2			3			4			5			6			7			8			Score
Parameter	N	D	S	N	D	S	N	D	S	N	D	S	N	D	S	N	D	S	N	D	S	N	D	S	
SJ01	87.5	97	90%	48	96.5	50%	91	96	95%	59	96	61%	73.5	96	77%	48	98	49%	61.5	98	63%	72.5	98	74%	69.8%
SJ02	83.5	97	86%	75	97	77%	76.5	97	79%	77	97	79%	82.5	97	85%	56	97	58%	66	97	68%	62.5	97	64%	74.6%
SJ03	88	96	92%	89	96	93%	93	96	97%	83	96	86%	87	96	91%	84.5	96	88%	87	96	91%	84	96	88%	90.6%
SJ03(2nd)	N/A	N/A	N/A	N/A	N/A	N/A	N/A	N/A	N/A	N/A	N/A	N/A	N/A	N/A	N/A	N/A	N/A	N/A	N/A	N/A	N/A	N/A	N/A	N/A	N/A
SJ04	76	95	80%	75	95	79%	73	95	77%	59.5	95	63%	67.5	95	71%	59	96	61%	52	96	54%	37	96	39%	65.5%
SJ04(2nd)	69.5	96	72%	55	96	57%	80	96	83%	51	96	53%	54.5	96	57%	53.5	96	56%	70	96	73%	31	96	32%	60.5%
SJ05	76	93	82%	79	93	85%	82	93	88%	70	93	75%	89	93	96%	87	93	94%	85	93	91%	79	93	85%	87.0%
SJ06	60	91	66%	91	91	100%	63	91	69%	73	91	80%	91	91	100%	87	91	96%	85	91	93%	81	91	89%	86.7%
SJ07	N/A	N/A	N/A	N/A	N/A	N/A	N/A	N/A	N/A	N/A	N/A	N/A	N/A	N/A	N/A	N/A	N/A	N/A	N/A	N/A	N/A	N/A	N/A	N/A	N/A
SJ08	N/A	N/A	N/A	N/A	N/A	N/A	N/A	N/A	N/A	N/A	N/A	N/A	N/A	N/A	N/A	N/A	N/A	N/A	N/A	N/A	N/A	N/A	N/A	N/A	N/A
Average			81%			77%			84%			71%			82%			72%			76%			67%	76.4%
StD			9.4%			18.1%			10.0%			12.2%			15.2%			19.9%			15.7%			23.4%	11.8%

Table S11. The PEQ prosthesis utility score for each participant using both passive liners and Roliner are tabulated below. The average score and standard deviation (StD) of prosthesis utility scores for passive liners and Roliner are shown at the end of each question columns. The difference between passive liner and Roliner for each participant are shown at the end of each row.

PEQ Utility Summary

	PEQ Standard	PEQ Roliner	Delta Δ
**SJ01**	74.5%	69.8%	-4.7%
**SJ02**	47.4%	74.6%	27.3%
**SJ03**	90.0%	90.6%	0.6%
**SJ03(2nd)**	72.0%	N/A	N/A
**SJ04**	13.1%	65.5%	52.4%
**SJ04(2nd)**	15.7%	60.5%	44.8%
**SJ05**	52.8%	87.0%	34.2%
**SJ06**	66.7%	86.7%	20.0%
**SJ07**	94.0%	N/A	N/A
**SJ08**	65.7%	N/A	N/A
**Average**	59.2%	76.4%	24.9%
**StD**	27.6%	11.8%	21.3%

Table S12. The prosthesis evaluation questionnaire (PEQ) scores of passive liners for questions related to residual limb health (questions 9-14) are tabulated below. The average score and standard deviation (StD) of each question are shown at the end of each question columns. The average score of each participant are shown at the end of each user column.

Residual Health (Standard)

Question #	9			10			11			12			13			14			Score
Parameter	N	D	S	N	D	S	N	D	S	N	D	S	N	D	S	N	D	S	
SJ01	75	97	77%	79	97	81%	65	97	67%	97	97	100%	97	97	100%	47	97	48%	79.0%
SJ02	53.5	97	55%	80.5	97	83%	56.5	97	58%	4.5	97	5%	90.5	97	93%	3.5	97	4%	49.7%
SJ03	70.5	96	73%	92.5	96	96%	96	96	100%	87.5	96	91%	83	96	86%	96	96	100%	91.2%
SJ03(2nd)	68	96.5	70%	96.5	96.5	100%	96.5	96.5	100%	83	96.5	86%	83	96.5	86%	96.5	96.5	100%	90.4%
SJ04	2	96	2%	14	96	15%	63.5	96	66%	96	96	100%	1	96	1%	96	96	100%	47.3%
SJ04(2nd)	2	93	2%	2	93	2%	57	93	61%	6	93	6%	6	93	6%	3	93	3%	13.6%
SJ05	21.5	96	22%	33	96	34%	35	96	36%	61	96	64%	91	96	95%	84	96	88%	56.5%
SJ06	57	96	59%	95	96	99%	94	96	98%	95	96	99%	95	96	99%	94	96	98%	92.0%
SJ07	66	93	71%	64	93	69%	88	93	95%	55	93	59%	93	93	100%	62	93	67%	76.7%
SJ08	11	91	12%	89	91	98%	88	91	97%	25	91	27%	77	91	85%	18.5	91	20%	56.5%
Average			45%			68%			78%			64%			75%			63%	65.3%
StD			31.2%			37.1%			22.7%			38.3%			38.1%			40.9%	25.2%

Table S13. The prosthesis evaluation questionnaire (PEQ) scores of Roliner for questions related to residual limb health (questions 9-14) are tabulated below. The average score and standard deviation (StD) of each question are shown at the end of each question columns. The average score of each participant are shown at the end of each user column.

Residual Health (Roliner)

Question #	9			10			11			12			13			14			Score
Parameter	N	D	S	N	D	S	N	D	S	N	D	S	N	D	S	N	D	S	
SJ01	83.5	98.5	85%	95.5	98.5	97%	89	97	92%	65	97	67%	97	97	100%	97	97	100%	90.1%
SJ02	85	97	88%	94.5	97	97%	73.5	97	76%	68	97	70%	97	97	100%	97	97	100%	88.5%
SJ03	79.5	96	83%	96	96	100%	96	96	100%	96	96	100%	96	96	100%	96	96	100%	97.1%
SJ03(2nd)	N/A	N/A	N/A	N/A	N/A	N/A	N/A	N/A	N/A	N/A	N/A	N/A	N/A	N/A	N/A	N/A	N/A	N/A	N/A
SJ04	15	96	16%	12.5	96	13%	59	96	61%	96	96	100%	96	96	100%	96	96	100%	65.0%
SJ04(2nd)	48	96	50%	47	96	49%	26	96	27%	45	96	47%	45	96	47%	47	96	49%	44.8%
SJ05	87	93	94%	87	93	94%	87	93	94%	81	93	87%	90	93	97%	88	93	95%	93.2%
SJ06	88	91	97%	89	91	98%	90	91	99%	90	91	99%	90	91	99%	89	91	98%	98.2%
	N/A	N/A	N/A	N/A	N/A	N/A	N/A	N/A	N/A	N/A	N/A	N/A	N/A	N/A	N/A	N/A	N/A	N/A	N/A
SJ08	N/A	N/A	N/A	N/A	N/A	N/A	N/A	N/A	N/A	N/A	N/A	N/A	N/A	N/A	N/A	N/A	N/A	N/A	N/A
Average			73%			78%			78%			81%			92%			92%	82.4%
StD			29.6%			34.0%			26.5%			20.6%			19.8%			18.9%	20.0%

Table S14. The PEQ residual limb health score for each participant using both passive liners and Roliner are tabulated below. The average score and standard deviation (StD) of prosthesis utility scores for passive liners and Roliner are shown at the end of each question columns. The difference between passive liner and Roliner for each participant are shown in the end of each row.

PEQ Residual Limb Health Summary

	PEQ Standard	PEQ Roliner	Delta Δ
**SJ01**	79.0%	90.1%	11.0%
**SJ02**	49.7%	88.5%	38.8%
**SJ03**	91.2%	97.1%	5.9%
**SJ03(2nd)**	90.4%	N/A	N/A
**SJ04**	47.3%	65.0%	17.7%
**SJ04(2nd)**	13.6%	44.8%	31.2%
**SJ05**	56.5%	93.2%	36.7%
**SJ06**	92.0%	98.2%	6.2%
**SJ07**	76.7%	N/A	N/A
**SJ08**	56.5%	N/A	N/A
**Average**	65.3%	82.4%	21.1%
**StD**	63.9%	20.0%	14.3%

Table S15. The prosthesis evaluation questionnaire (PEQ) scores of passive liners for questions related to frustration (questions 15-16) are tabulated below. The average score and standard deviation (StD) of each question are shown at the end of each question columns. The average score of each participant are shown at the end of each user column.

Frustration (Standard)

Question #	15			16			Score
Parameter	N	D	S	N	D	S	
SJ01	62	97	64%	7	97	7%	35.6%
SJ02	15	97	15%	1.5	97	2%	8.5%
SJ03	87	96	91%	96	96	100%	95.3%
SJ03(2nd)	79.5	96.5	82%	12.5	96.5	13%	47.7%
SJ04	0	96	0%	0	96	0%	0.0%
SJ04(2nd)	0	96	0%	0	96	0%	0.0%
SJ05	74.5	96	78%	74.5	96	78%	77.6%
SJ06	86	96	90%	76	96	79%	84.4%
SJ07	92	93	99%	93	93	100%	99.5%
SJ08	29	91	32%	6	91	7%	19.2%
Average			55%			39%	46.8%
StD			39.3%			44.4%	46.8%

Table S 16. The prosthesis evaluation questionnaire (PEQ) scores of Roliner for questions related to frustration (questions 15-16) are tabulated below. The average score and standard deviation (StD) of each question are shown at the end of each question columns. The average score of each participant is shown at the end of each user column.

Frustration (Roliner)

Question #	15			16			Score
Parameter	N	D	S	N	D	S	
SJ01	70	97	72%	97	97	100%	86.1%
SJ02	89	97	92%	97	97	100%	95.9%
SJ03	91	96	95%	96	96	100%	97.4%
SJ03(2nd)	N/A	N/A	N/A	N/A	N/A	N/A	N/A
SJ04	66	96	69%	96	96	100%	84.4%
SJ04(2nd)	70	96	73%	53	96	55%	64.1%
SJ05	87	93	94%	87	93	94%	93.5%
SJ06	89	91	98%	80	91	88%	92.9%
SJ07	N/A	N/A	N/A	N/A	N/A	N/A	N/A
SJ08	N/A	N/A	N/A	N/A	N/A	N/A	N/A
Average			85%			91%	87.7%
StD			12.6%			16.4%	14.5%

Table S17. The PEQ frustration score for each participant using both passive liners and Roliner are tabulated below. The average score and standard deviation (StD) of prosthesis utility scores for passive liners and Roliner are shown at the end of each question columns. The difference between passive liner and Roliner for each participant are shown at the end of each row.

PEQ Frustration Summary

	PEQ Standard	PEQ Roliner	Delta Δ
**SJ01**	35.6%	86.1%	50.5%
**SJ02**	8.5%	95.9%	87.4%
**SJ03**	95.3%	97.4%	2.1%
**SJ03(2nd)**	47.7%	N/A	N/A
**SJ04**	0.0%	84.4%	84.4%
**SJ04(2nd)**	0.0%	64.1%	64.1%
**SJ05**	77.6%	93.5%	15.9%
**SJ06**	84.4%	92.9%	8.5%
**SJ07**	99.5%	N/A	N/A
**SJ08**	46.8%	N/A	N/A
**Average**	49.5%	87.7%	44.7%
**StD**	38.6%	11.5%	36.0%

Table S18. The prosthesis evaluation questionnaire (PEQ) scores of passive liners for questions related to ambulation (questions 17-24) are tabulated below. The average score and standard deviation (StD) of each question are shown at the end of each question columns. The average score of each participant are shown at the end of each user column.

Ambulation (Standard)

Question #	17			18			19			20			21			22			23			24			Score
**Parameter**	**N**	**D**	**S**	**N**	**D**	**S**	**N**	**D**	**S**	**N**	**D**	**S**	**N**	**D**	**S**	**N**	**D**	**S**	**N**	**D**	**S**	**N**	**D**	**S**	
**SJ01**	78	98.5	**79%**	88.5	98.5	**90%**	12.5	98.5	**13%**	13	98.5	**13%**	54.5	99	**55%**	44.5	99.5	**45%**	89	97.5	**91%**	4	98	**4%**	**48.8%**
**SJ02**	45.5	97	**47%**	41.5	97	**43%**	41.5	97	**43%**	56.5	97	**58%**	37.5	97	**39%**	38	97	**39%**	54.5	97	**56%**	29	97	**30%**	**44.3%**
**SJ03**	86.5	96	**90%**	87.5	96	**91%**	89	96	**93%**	87	96	**91%**	87	96	**91%**	87	96	**91%**	88	96	**92%**	88.5	96	**92%**	**91.2%**
**SJ03(2nd)**	86	96.5	**89%**	64.5	96.5	**67%**	65	96.5	**67%**	62	96.5	**64%**	37.5	96.5	**39%**	27	96.5	**28%**	83	96.5	**86%**	66	96.5	**68%**	**63.6%**
**SJ04**	29	96	**30%**	17	96	**18%**	21	96	**22%**	27	96	**28%**	27	96	**28%**	25	96	**26%**	24	96	**25%**	14	96	**15%**	**24.0%**
**SJ04(2nd)**	18	93	**19%**	5	93	**5%**	5	93	**5%**	13	93	**14%**	16.5	93	**18%**	15.5	93	**17%**	13.5	93	**15%**	15	93	**16%**	**13.6%**
**SJ05**	65	96	**68%**	66	96	**69%**	69.5	96	**72%**	74.5	96	**78%**	75.5	96	**79%**	45.5	96	**47%**	63	96	**66%**	39	96	**41%**	**64.8%**
**SJ06**	92	96	**96%**	75	96	**78%**	6	96	**6%**	92	96	**96%**	84	96	**88%**	94	96	**98%**	84	96	**88%**	92	96	**96%**	**80.6%**
**SJ07**	92	93	**99%**	81	93	**87%**	88	93	**95%**	86	93	**92%**	92	93	**99%**	80	93	**86%**	92	93	**99%**	80	93	**86%**	**92.9%**
**SJ08**	72	91	**79%**	73	91	**80%**	21	91	**23%**	45	91	**49%**	48	91	**53%**	49	91	**54%**	67	91	**74%**	2	91	**2%**	**51.8%**
**Average**			**70%**			**63%**			**44%**			**58%**			**59%**			**53%**			**69%**			**45%**	**57.6%**
**StD**			**28.1%**			**30.6%**			**35.1%**			**31.7%**			**28.5%**			**28.8%**			**29.1%**			**37.3%**	**26.5%**

Table S19. The prosthesis evaluation questionnaire (PEQ) scores of Roliner for questions related to ambulation (questions 17-24) are tabulated below. The average score and standard deviation (StD) of each question are shown at the end of each question columns. The average score of each participant is shown at the end of each user column.

Ambulation (Roliner)

Question #	17			18			19			20			21			22			23			24			Score
Parameter	N	D	S	N	D	S	N	D	S	N	D	S	N	D	S	N	D	S	N	D	S	N	D	S	
SJ01	72	95	76%	73.5	94.5	78%	21	94.5	22%	19	94.5	20%	59.5	95.5	62%	34	95	36%	75	94.5	79%	2	94.5	2%	46.9%
SJ02	87	97	90%	94	97	97%	59	97	61%	91	97	94%	88.5	97	91%	86.5	97	89%	92.5	97	95%	73.5	97	76%	86.6%
SJ03	90.5	96	94%	88.5	96	92%	89	96	93%	88	96	92%	85.5	96	89%	87	96	91%	86	96	90%	84	96	88%	91.0%
SJ03(2nd)	N/A	N/A	N/A	N/A	N/A	N/A	N/A	N/A	N/A	N/A	N/A	N/A	N/A	N/A	N/A	N/A	N/A	N/A	N/A	N/A	N/A	N/A	N/A	N/A	N/A
SJ04	78	96	81%	68	96	71%	67	96	70%	69	96	72%	71	96	74%	64	96	67%	68.5	96	71%	70	96	73%	72.3%
SJ04(2nd)	69.5	96	72%	52.5	96	55%	57	96	59%	58.5	96	61%	58	96	60%	54	96	56%	55	96	57%	41	96	43%	58.0%
SJ05	87	93	94%	88	93	95%	86	93	92%	83	93	89%	83	93	89%	84	93	90%	84	93	90%	80	93	86%	90.7%
SJ06	90	91	99%	90	91	99%	66	91	73%	89	91	98%	74	91	81%	90	91	99%	82	91	90%	16	91	18%	82.0%
SJ07	N/A	N/A	N/A	N/A	N/A	N/A	N/A	N/A	N/A	N/A	N/A	N/A	N/A	N/A	N/A	N/A	N/A	N/A	N/A	N/A	N/A	N/A	N/A	N/A	N/A
SJ08	N/A	N/A	N/A	N/A	N/A	N/A	N/A	N/A	N/A	N/A	N/A	N/A	N/A	N/A	N/A	N/A	N/A	N/A	N/A	N/A	N/A	N/A	N/A	N/A	N/A
Average			87%			84%			67%			75%			78%			75%			82%			55%	75.4%
StD			10.1%			16.5%			24.0%			27.6%			13.0%			23.1%			13.6%			34.4%	17.2%

Table S20. The PEQ ambulation score for each participant using both passive liners and Roliner are tabulated below. The average score and standard deviation (StD) of prosthesis utility scores for passive liners and Roliner are shown at the end of each question columns. The difference between passive liner and Roliner for each participant are shown at the end of each row.

PEQ Ambulation Summary

	PEQ Standard	PEQ Roliner	Delta Δ
SJ01	48.8%	46.9%	-1.8%
SJ02	44.3%	86.6%	42.3%
SJ03	91.2%	91.0%	-0.3%
SJ03(2nd)	63.6%	N/A	N/A
SJ04	24.0%	72.3%	48.4%
SJ04(2nd)	13.6%	58.0%	44.4%
SJ05	64.8%	90.7%	25.9%
SJ06	80.6%	82.0%	1.4%
SJ07	92.9%	N/A	N/A
SJ08	51.8%	N/A	N/A
Average	57.6%	75.4%	22.9%
StD	26.5%	17.2%	22.7%

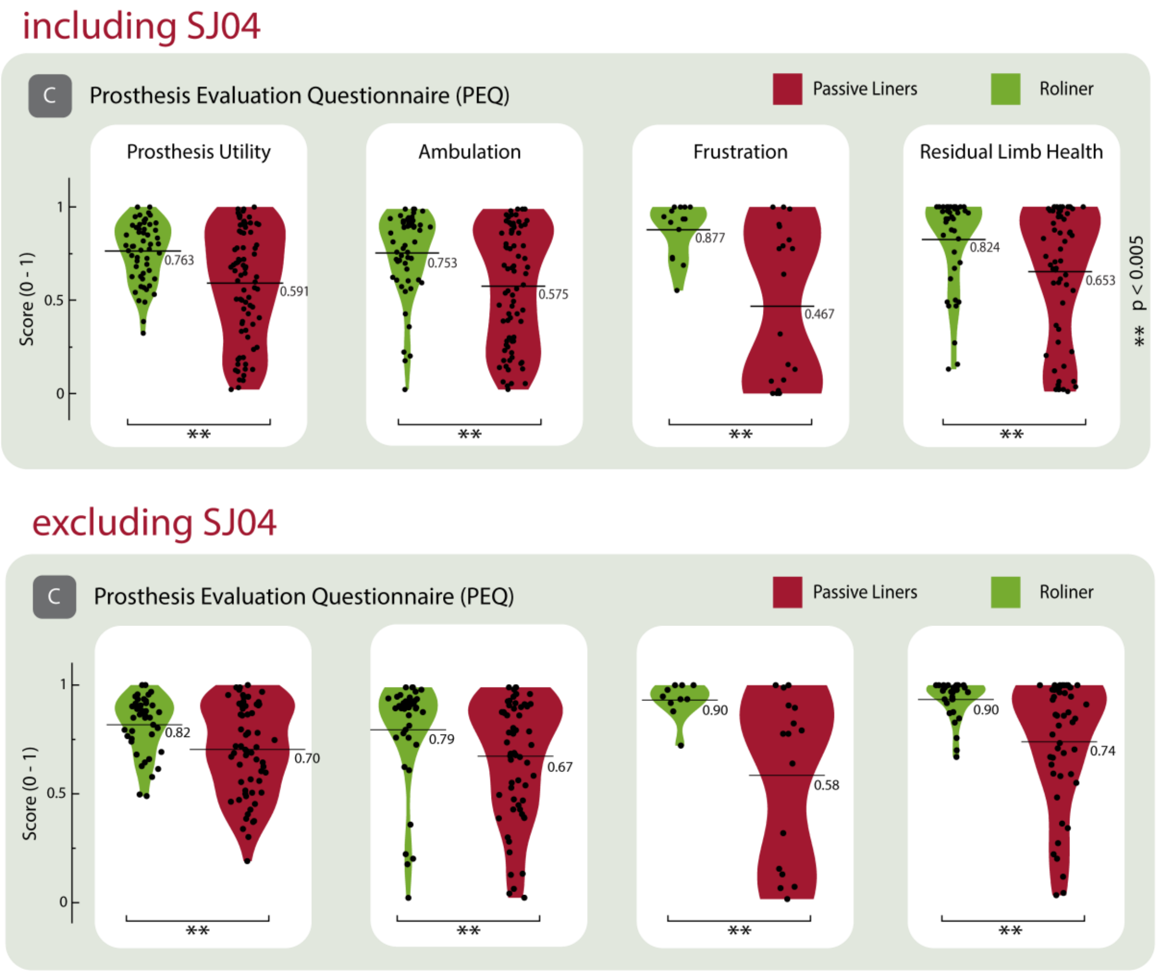

Fig. S14. The violin chart comparison of Roliner versus passive liners in terms of prosthetic utility, ambulation, frustration and residual limb health parameters Prosthesis Evaluation Questionnaire (PEQ) scores from the preclinical cohort (except SJ04, removed for potential bias). The scores from each question response from each amputee are shown as black-filled circles. The horizontal line shows the mean score. The mean scores (μ) are shown on the right-hand side of the violin chart. In the violin charts, kernel density estimation was performed using Scott type bandwidth. The individual data points represents the independent PEQ scores from the related section ($8 \geq n \geq 2$) of the questionnaire for each participant ($n = 6$). These were presented as overlayed on the box charts. The p value calculated using two-sided paired sample t-test. (**) represents statistical significance as $p < 0.005$.
