# Supplementary material for: Dynamically adaptive soft metamaterial for wearable human–machine interfaces
Source: Nat Commun. 2025 Mar 19;16:2621. doi: 10.1038/s41467-025-57634-8 (PMC11923287; doi:10.1038/s41467-025-57634-8)
Supplement: Supplementary file 2 — Description of Additional Supplementary Files [file 41467_2025_57634_MOESM2_ESM.pdf]

Description of Additional Supplementary Files

Supplementary Movie 1 Circular Channel Millifluidics

Supplementary Movie 2 Hexagonal Channel Millifluidics

Supplementary Movie 3 Roliner First Fitting Trial - SJ04

Supplementary Movie 4 Motion Lab Gait Tracking - SJ03
