# Supplementary material for: Dynamically adaptive soft metamaterial for wearable human–machine interfaces
Source: Nat Commun. 2025 Mar 19;16:2621. doi: 10.1038/s41467-025-57634-8 (PMC11923287; doi:10.1038/s41467-025-57634-8)
Supplement: Supplementary file 7 — Reporting Summary [file 41467_2025_57634_MOESM7_ESM.pdf]

nature portfolio

Corresponding author(s): Firat Güder

Last updated by author(s): Jan 20, 2025

Reporting Summary

Nature Portfolio wishes to improve the reproducibility of the work that we publish. This form provides structure for consistency and transparency in reporting. For further information on Nature Portfolio policies, see our Editorial Policies and the Editorial Policy Checklist.

Statistics

For all statistical analyses, confirm that the following items are present in the figure legend, table legend, main text, or Methods section.

n/a	Confirmed	
[ ]	[x]	The exact sample size (*n*) for each experimental group/condition, given as a discrete number and unit of measurement
[ ]	[x]	A statement on whether measurements were taken from distinct samples or whether the same sample was measured repeatedly
[ ]	[x]	The statistical test(s) used AND whether they are one- or two-sided<br>*Only common tests should be described solely by name; describe more complex techniques in the Methods section.*
[x]	[ ]	A description of all covariates tested
[x]	[ ]	A description of any assumptions or corrections, such as tests of normality and adjustment for multiple comparisons
[ ]	[x]	A full description of the statistical parameters including central tendency (e.g. means) or other basic estimates (e.g. regression coefficient) AND variation (e.g. standard deviation) or associated estimates of uncertainty (e.g. confidence intervals)
[ ]	[x]	For null hypothesis testing, the test statistic (e.g. *F*, *t*, *r*) with confidence intervals, effect sizes, degrees of freedom and *P* value noted<br>*Give P values as exact values whenever suitable.*
[x]	[ ]	For Bayesian analysis, information on the choice of priors and Markov chain Monte Carlo settings
[x]	[ ]	For hierarchical and complex designs, identification of the appropriate level for tests and full reporting of outcomes
[x]	[ ]	Estimates of effect sizes (e.g. Cohen's *d*, Pearson's *r*), indicating how they were calculated

Our web collection on statistics for biologists contains articles on many of the points above.

Software and code

Policy information about availability of computer code

Data collection	Data collection for mechanical measurements was performed using Mecmesin Emperor™ Force and Torque Testing Software (v1.18-408, Mecmesin Ltd.), the Roliner actuation was performed using Roliner app (v15.0., Unhindr Ltd.). The data collection for gait cycle analysis was performed using Vicon Nexus (v2.12, Vicon Motion Systems Ltd.)
Data analysis	The data analysis for mechanical measurements was performed using Mecmesin Emperor™ Force and Torque Testing Software (1.18-408, Mecmesin Ltd.). The gait cycle parameter analysis was performed using Vicon Nexus (v2.12, Vicon Motion Systems Ltd.). The visualization of the data was performed using MATLAB R2023a (v9.14.0.2206163

For manuscripts utilizing custom algorithms or software that are central to the research but not yet described in published literature, software must be made available to editors and reviewers. We strongly encourage code deposition in a community repository (e.g. GitHub). See the Nature Portfolio guidelines for submitting code & software for further information.

Data

Policy information about availability of data

All manuscripts must include a data availability statement. This statement should provide the following information, where applicable:

- Accession codes, unique identifiers, or web links for publicly available datasets
- A description of any restrictions on data availability
- For clinical datasets or third party data, please ensure that the statement adheres to our policy

The datasets generated during and/or analyzed during the current study are available from the corresponding author upon reasonable request.

Research involving human participants, their data, or biological material

Policy information about studies with human participants or human data. See also policy information about sex, gender (identity/presentation), and sexual orientation and race, ethnicity and racism.

Reporting on sex and gender	The sex of the individual participants were listed for the manuscript. We do not draw into any conclusions regarding the sex of the patients.
Reporting on race, ethnicity, or other socially relevant groupings	The study does not have any form of information regarding race, ethnicity, or other socially relevant groups.
Population characteristics	The population characteristics is beyond the scope of this study. We do not draw into any conclusions regarding population characteristics.
Recruitment	The recruitment was implemented using advertisements. The volunteers were screened using the inclusion and exclusion criteria.<br><br>Inclusion Criteria<br>The amputees are selected from diverse ethnic and social backgrounds. The amputee must:<br>• be a lower-limb amputee between 18 and 60 years<br>• be a healthy adult with no other medical conditions,<br>• have been an amputee for more than 18 months, traumatic or surgically induced.<br><br>Exclusion Criteria<br>Members of vulnerable groups are excluded (e.g., those under 18, prisoners, those in a dependent relationship, the mentally ill). Additionally, the amputee must not:<br>• have had diabetic or other vascular-related amputations,<br>• be taking any medication or receiving any therapy for another existing condition<br>• have any allergies to adhesive materials<br>• have undergone surgery in the lower limbs in the 12 months prior to participation<br>• be unable to speak English at a sufficient level to give consent to participate<br>• be pregnant<br>• be younger than 18 or older than 60 years of age<br>• weigh above 120 kg in mass
Ethics oversight	This pre-clinical trial was approved by Research Governance and Integrity Team (RGIT) under Imperial College Research Ethics Committee process (ICREC Ref No: 21IC7315) in December 2nd, 2021.

Note that full information on the approval of the study protocol must also be provided in the manuscript.

Field-specific reporting

Please select the one below that is the best fit for your research. If you are not sure, read the appropriate sections before making your selection.

- [x] Life sciences
- [ ] Behavioural & social sciences
- [ ] Ecological, evolutionary & environmental sciences

For a reference copy of the document with all sections, see nature.com/documents/nr-reporting-summary-flat.pdf

Life sciences study design

All studies must disclose on these points even when the disclosure is negative.

Sample size	No sample size calculation was performed in this pre-clinical study.
Data exclusions	No data were excluded in the initial submission.

Replication	The measurements were recorded multiple times to construct the statistical characteristics (i.e. SD)
Randomization	not applicable.
Blinding	not applicable, our focus was on the non-inferiority of the proposed technology.

Reporting for specific materials, systems and methods

We require information from authors about some types of materials, experimental systems and methods used in many studies. Here, indicate whether each material, system or method listed is relevant to your study. If you are not sure if a list item applies to your research, read the appropriate section before selecting a response.

Materials & experimental systems

n/a	Involved in the study
☒	☐ Antibodies
☒	☐ Eukaryotic cell lines
☒	☐ Palaeontology and archaeology
☒	☐ Animals and other organisms
☒	☐ Clinical data
☒	☐ Dual use research of concern
☒	☐ Plants

Methods

n/a	Involved in the study
☒	☐ ChIP-seq
☒	☐ Flow cytometry
☒	☐ MRI-based neuroimaging

Plants

Seed stocks	*Report on the source of all seed stocks or other plant material used. If applicable, state the seed stock centre and catalogue number. If plant specimens were collected from the field, describe the collection location, date and sampling procedures.*
Novel plant genotypes	*Describe the methods by which all novel plant genotypes were produced. This includes those generated by transgenic approaches, gene editing, chemical/radiation-based mutagenesis and hybridization. For transgenic lines, describe the transformation method, the number of independent lines analyzed and the generation upon which experiments were performed. For gene-edited lines, describe the editor used, the endogenous sequence targeted for editing, the targeting guide RNA sequence (if applicable) and how the editor was applied.*
Authentication	*Describe any authentication procedures for each seed stock used or novel genotype generated. Describe any experiments used to assess the effect of a mutation and, where applicable, how potential secondary effects (e.g. second site T-DNA insertions, mosiacism, off-target gene editing) were examined.*
